# A case of hyperparathyroidism-associated parkinsonism successfully treated with cinacalcet hydrochloride, a calcimimetic

**DOI:** 10.1186/s12883-018-1067-7

**Published:** 2018-05-07

**Authors:** Yuichiro Ohya, Masato Osaki, Shota Sakai, Shunsuke Kimura, Chiharu Yasuda, Tetsuro Ago, Takanari Kitazono, Shuji Arakawa

**Affiliations:** 1 Department of Cerebrovascular Medicine, Stroke Center, Steel Memorial Yawata Hospital, Kitakyushu, Japan; 2 Department of Medicine and Clinical Science, Graduate School of Medical Sciences, Kyushu University, Fukuoka, Japan; 3 Department of Cerebrovascular Medicine, Kyushu Central Hospital, 3-23-1 Shiobaru, Minami-ku, Fukuoka-shi, Fukuoka 815-8588 Japan

**Keywords:** Cinacalcet, Hypercalcemia, Hyperparathyroidism, Parkinsonism

## Abstract

**Background:**

Some metabolic disorders, including abnormal calcium metabolism, can develop and worsen parkinsonism. However, whether hyperparathyroidism can cause parkinsonism remains controversial.

**Case presentation:**

An 83-year-old woman with a history of right thalamic hemorrhage and drug-induced parkinsonism, was admitted due to worsening of parkinsonian symptoms including mask-like face, bradykinesia, freezing of gait, and rigidity. She had been diagnosed with autoimmune hepatitis and was being treated with prednisolone. Examinations revealed hypercalcemia (14.3 mg/dL) with an increased level of intact parathyroid hormone (iPTH) (361 pg/mL). Her symptoms were resistant to some additional anti-parkinsonian drugs; however, cinacalcet hydrochloride, a calcimimetic for the treatment of secondary hyperparathyroidism, normalized levels of serum calcium and iPTH, and remarkably improved her symptoms.

**Conclusions:**

In the present case, we speculate that hypercalcemia probably due to secondary hyperparathyroidism that had developed during steroid therapy deteriorated the parkinsonism.

## Background

Parkinson’s disease is a degenerative disorder that affects the neuromotor system and is characterized by extrapyramidal symptoms including bradykinesia, rigidity, tremor, postural instability, and the freezing phenomenon. The loss of dopamine-secreting neurons in the substantia nigra induces a relative deficiency of dopamine and imbalance of dopamine and other various neurotransmitters including acetylcholine, glutamate, and GABA in the basal ganglia. Secondary parkinsonism is a disease state in which actions of dopamine in the basal ganglia is blocked or interfered by drugs, toxicants, cerebrovascular diseases, metabolic disorders and various comorbidities. Although hypoparathyroidism is known to cause secondary parkinsonism, whether hyperparathyroidism can cause secondary parkinsonism remains controversial [1–6]. We describe an 83-year-old woman with hyperparathyroidism whose deteriorated parkinsonian symptoms were successfully treated with cinacalcet hydrochloride, a calcimimetic used for the treatment of hyperparathyroidism, while her neurological symptoms had been resistant to anti-parkinsonian drugs.

## Case presentation (Fig. 1)

An 83-year-old woman was admitted to our hospital because of uncontrolled parkinsonism. She had been diagnosed with autoimmune hepatitis at 40 years of age and intermittently treated with prednisolone. She had developed right thalamic hemorrhage at the age of 73 years. Although she had developed risperidone-induced parkinsonism at 79 years of age, discontinuation of the risperidone and administration of L-dopa (600 mg/day) improved her symptoms. The Unified Parkinson’s Disease Rating Scale (UPDRS) based on the information from her previous doctor was assumed to improve from around 10 to nearly 0 after the discontinuation of risperidone and administration of L-dopa.

**Fig. 1:**
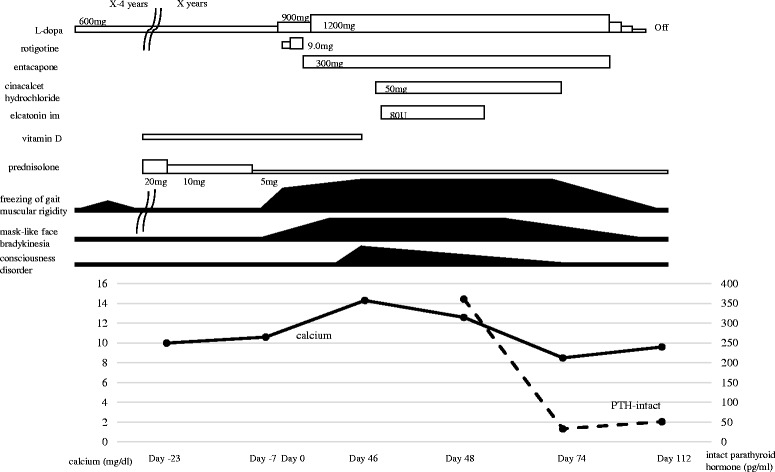
The patient’s clinical course before and after the administration of cinacalcet hydrochloride. The levels of serum calcium and intact parathyroid hormone declined rapidly after the treatment

One month before the current admission, prednisolone (20 mg/day) had been re-stared because of the recurrence of autoimmune hepatitis. During the tapering of prednisolone from 20 to 5 mg/day within 2 weeks, her parkinsonian symptoms, including mask-like face, bradykinesia, freezing of gait, and rigidity, began to worsen. Her parkinsonian symptoms had worsened to 57 points on the UPDRS by the time of admission. She became unable to walk and her verbal response declined, although her conscious level remained clear. The symptoms were resistant to an increased dosage of L-dopa from 600 to 1200 mg/day as well as the additional administration of a dopamine receptor agonist and catechol-O-methyltransferase inhibitor.

**Fig. 2:**
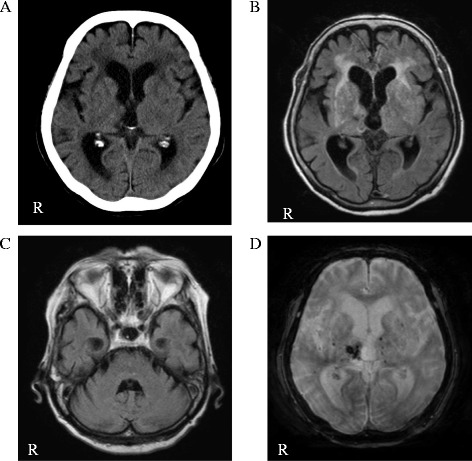
**a**. Brain computed tomography did not show basal ganglia calcification. **b.** Magnetic resonance imaging fluid-attenuated inversion recovery showed slit hyperintensity of the lateral margin of the right putamen but did not show appreciable atrophy of the basal ganglia. **c.** Magnetic resonance imaging fluid-attenuated inversion recovery did not show appreciable atrophy of the pons and cerebellum. **d**. T2* magnetic resonance imaging showed an old intracranial hemorrhage in the right thalamus. “R” indicates the right side

**Fig. 3:**
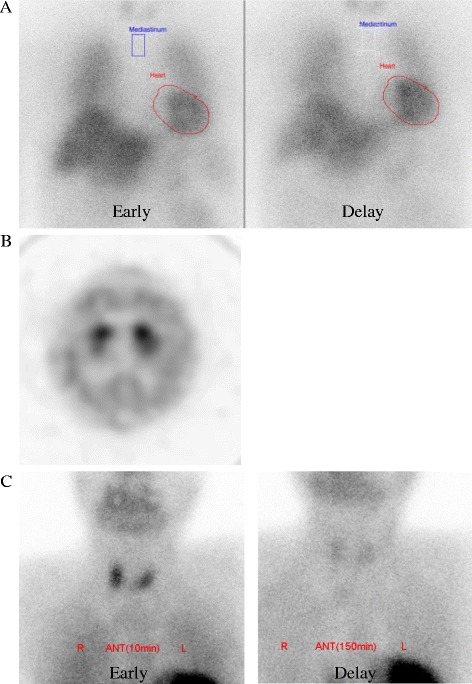
**a**. 123I-metaiodobenzylguanidine myocardial scintigraphy did not show a low heart/superior mediastinum ratio or acceleration of the washout rate (early and delayed heart/superior mediastinum ratio of 2.54 and 2.76, washout rate of − 5.4%). **b**. Dopamine transporter scan did not show a decrease in striatal accumulation. **c**. ^99m^Tc methoxy-isobutyl-isonitrile scintigraphy did not show abnormal accumulation in the parathyroid

Brain computed tomography, magnetic resonance imaging, and 123I-metaiodobenzylguanidine myocardial scintigraphy revealed no abnormalities that could cause parkinsonism (Figs. 2 and 3a). There was no decrease in the uptake of dopamine transporters in the striatum on the dopamine transporter scan (Fig. 3b).

At 46 days after admission, the patient’s conscious level decreased to 9 points (E2, V3, M4) on the Glasgow coma scale. Detailed evaluation of her parkinsonism was difficult because of her decreased consciousness. The level of total serum calcium, corrected by albumin, was remarkably elevated at 14.3 mg/dL with an increased level of intact parathyroid hormone (iPTH) (361 pg/mL). Computed tomography and ultrasound scans showed no enlargement of the parathyroid gland. ^99m^Tc methoxy-isobutyl-isonitrile scintigraphy showed no increased accumulation in the parathyroid (Fig. 3c). To correct the hypercalcemia with the increased iPTH, we administered cinacalcet hydrochloride, a calcimimetic for the treatment of secondary hyperparathyroidism. Three weeks after commencing cinacalcet, the patient’s total serum calcium normalized to 8.5 mg/dL with a normal iPTH level (33 pg/mL). Along with the normalization of the serum calcium and iPTH, both her conscious level and parkinsonian symptoms gradually improved. She became able to stand and walk without support. Her UPDRS score recovered to 7 points while slight bradykinesia remained, which may be due to her advanced age. Even after discontinuing the anti-parkinsonian drugs, her neurological conditions were stable. Furthermore, the levels of total serum calcium and iPTH remained within normal limits 1 month after discontinuance of cinacalcet.

## Discussion

We have reported a case of secondary parkinsonism associated with increased levels of calcium and iPTH that developed during steroid therapy for autoimmune hepatitis. The clinical symptoms were drastically improved by the correction of hypercalcemia, although these symptoms were resistant to anti-parkinsonian drugs. We consider that the secondary hyperparathyroidism may have been crucially involved in the deterioration of the patient’s parkinsonism based on her clinical course.

Hypoparathyroidism with calcification of the basal ganglia is well-recognized as a cause of secondary parkinsonism [2]. However, secondary parkinsonism associated with hyperparathyroidism is rare, and only a few such cases have been reported [1, 3–6]. Table 1 summarizes the clinical features of hyperparathyroidism-associated parkinsonism in eight reported cases and ours. Only our case involved secondary hyperparathyroidism; the other eight cases involved primary hyperparathyroidism. All patients showed hypercalcemia (> 11 mg/dL). Like hypoparathyroidism, calcification of the basal ganglia was found in two patients. The parkinsonian symptoms were relieved by the treatment of hyperparathyroidism in three patients, including ours, while most patients did not respond to anti-parkinsonian drugs as did not ours. To the best of our knowledge, the herein-described patient is the first to be successfully treated with cinacalcet hydrochloride for hyperparathyroidism-associated parkinsonism. Cinacalcet hydrochloride is a calcimimetic used for the treatment of secondary hyperparathyroidism, particularly under conditions of chronic renal failure, by activating calcium-sensing receptors to suppress the release of iPTH from the parathyroid [7–9]. Based on the clinical course, the present case clearly supports the concept that hyperparathyroidism-related hypercalcemia can worsen parkinsonism.

**Table 1:** Reported cases of parkinsonism associated with hyperparathyroidism

Patient	Authors	Age/ Sex	Serum Ca level (mg/dL)	Basal ganglia calcification	Response to antiparkinson drugs	Subtype of hyperparathyroidism	Treatment for hyperparthyroidism	Effect of treatment for hyperparathyroidism
1	Margolin D, et al. [3]	55/F	11.3	Yes	Poor	Primary	No treatment	No treatment
2	Margolin D, et al. [3]	84/F	11.8	Yes	Poor	Primary	Parathyroidectomy	No improvement
3	Müller R, et al. [4]	44/F	12.2	No	N.D.	Primary	Parathyroidectomy	No improvement
4	Hirooka Y, et al. [5]	70/F	11.6	No	Poor	Primary	Parathyroidectomy	Improved
5	Kovacs CS, et al. [6]	74/F	11.1	No	Poor	Primary	Parathyroidectomy	Improved
6	De Rosa A, et al. [1]	45/F	14.5	No	N.D.	Primary	Parathyroidectomy	No improvement
7	De Rosa A, et al. [1]	42/F	11.1	No	Good	Primary	N.D.	N.D.
8	De Rosa A, et al. [1]	65/M	11.8	No	Good	Primary	N.D.	N.D.
9	Present case	83/F	14.3	No	Poor	Secondary	Cinacalcet hydrochloride	Improved

*Ca* Calcium, *N.D* Not described, *F* Female, *M* Male

There are various possible mechanisms by which cinacalcet hydrochloride improved the present parkinsonism. First, hypercalcemia is thought to result in the release of a large quantity of dopamine into the synaptic cleft [10]. High concentrations of dopamine in the synaptic cleft may cause downregulation of postsynaptic dopamine receptors, leading to parkinsonism [11]. Second, iPTH reportedly induces neurotoxicity through induction of apoptotic neuronal death, independent of hypercalcemia [3, 5, 12]. Thus, the normalization of hypercalcemia and/or increased iPTH level by cinacalcet hydrochloride may have contributed to the improvement in the parkinsonism of our patient by preventing complete neuronal death.

Because steroids suppress calcium absorption from the intestinal tract and calcium reabsorption from the renal tubules, steroid treatment can cause secondary hyperparathyroidism in a compensatory manner. We speculate that rapid reduction of the steroid dose can cause hypercalcemia when the compensatory hyperparathyroidism is not immediately canceled. The fact that we could discontinue the cinacalcet without the recurrence of secondary hyperparathyroidism and parkinsonism in the chronic phase of the present case may support our speculation. The steroid therapy may have been the key factor in the close association between hyperparathyroidism and parkinsonism in the present case.

## Conclusion

In conclusion, we have reported a case of parkinsonism that was probably worsened by secondary hyperparathyroidism occurring during steroid therapy. While the parkinsonism was resistant to anti-parkinsonian drugs, cinacalcet hydrochloride remarkably relieved the parkinsonism along with correction of the hypercalcemia and the increased iPTH level. Physicians should note that calcium metabolism can affect parkinsonian symptoms through maladaptive parathyroid functions.

## Abbreviations

iPTH: Intact parathyroid hormone
UPDRS: Unified Parkinson’s Disease Rating Scale
